# Effect of lipid-lowering therapies on flow-mediated dilation in patients: A systematic review and meta-analysis of clinical randomized controlled trials

**DOI:** 10.1371/journal.pone.0323210

**Published:** 2025-06-03

**Authors:** Xinyue Wang, Lijun Zhou, Qiutao Wang, Min Wu

**Affiliations:** Guang’an Men Hospital, China Academy of Chinese Medical Sciences, Beijing, China; Università degli Studi di Milano: Universita degli Studi di Milano, ITALY

## Abstract

Numerous lipid-lowering medications are commonly used in clinical settings; however, their impact on vascular endothelial function remains unclear. This study employed techniques like flow-mediated dilation (FMD) to demonstrate the relative effects of lipid-lowering medications on vascular function. PubMed, Embase, and World of Science were searched from January 1, 2011 to October 1, 2024, and the language was limited to English. Randomized controlled trials (RCTs) have assessed the impact of lipid-lowering medications versus placebos on FMD in individuals. The outcomes included FMD, pulse wave velocity (PWV), low-density lipoprotein cholesterol (LDL-C), peak O_2_ consumption (VO_2_), and intimal media thickness (IMT). We computed standardized mean differences and 95% confidence intervals (CIs). P < 0.05 indicates statistical significance. The quality of the RCTs was assessed according to the methods provided by the Cochrane Handbook, and effective data were extracted. Revman software 5.4 version was used for statistical analysis. Drug type, intervention duration, and underlying diseases were used as covariates in the subgroup analysis. This meta-analysis included 19 RCTs involving 1,004 patients. Compared with placebo, lipid-lowering agents significantly reduced FMD (0.20 [95% CI: 0.05, 0.35], P = 0.007, I^2^ = 43%, 14 trials, 726 participants), LDL-C (−1.54 [95% CI: −1.78, −1.30], P < 0.00001, I^2 ^= 25%, 7 trials, 350 participants) and PWV (−0.35 [95% CI: −0.57, −0.02], P = 0.04, I^2 ^= 0.0%, 4 trials, 206 participants). Lipid-lowering drugs positively affect endothelial function, while lowering blood lipids and statins are the most effective.

## Background

Cardiovascular diseases are the primary cause of illness and death worldwide and are responsible for approximately 17.9 million deaths annually [1]. Atherosclerosis, a chronic inflammatory condition affecting the arterial wall, is an underlying factor in many cardiovascular disorders, such as coronary artery disease and stroke. The progression of atherosclerosis is affected by various risk factors, such as dyslipidemia, hypertension, smoking, and diabetes, making early identification and effective management crucial to reducing cardiovascular events [2].

Lipid-lowering therapies have demonstrated robust efficacy in mitigating atherosclerotic progression by reducing serum lipid levels. Many clinical trials have confirmed that lipid-lowering therapies play a pivotal role in cholesterol biosynthesis and are vital for secondary prevention [3], effectively lowering the risk of recurrent events in patients with established cardiovascular diseases.

Endothelial function is a key component of cardiovascular health, which can be quantitatively measured using FMD [4]. FMD is a non-invasive technique that assesses the vasodilatory reaction of the brachial artery in response to elevated blood flow and reflects endothelial nitric oxide (NO) production, a key indicator of vascular health [5]. Lipid-lowering therapies have pleiotropic effects, such as enhancing endothelial function by increasing the availability of NO. They achieve this by upregulating endothelial nitric oxide synthase, which leads to increased production—a key mediator of vascular relaxation and inhibition of inflammation and thrombosis [6]. Additionally, statins decrease oxidative stress by inhibiting the generation of reactive oxygen species, which degrade NO and preserve its vasodilatory function. Other lipid-lowering agents, such as PCSK9 inhibitors, have also been shown to improve FMD, likely through similar mechanisms involving improved lipid profiles and decreased vascular inflammation [7]. Reduced FMD is an early indicator of endothelial dysfunction and can predict future cardiovascular events, making it a valuable tool for early diagnosis and risk stratification. Studies have reported that lipid-lowering interventions can improve FMD, potentially reversing endothelial dysfunction and contributing to a reduced cardiovascular risk [8]. Lipid-lowering therapies improve FMD through several mechanisms, primarily associated with their impact on endothelial function and NO production.

However, their effects on FMD, a marker of endothelial function, have inconsistent results in different studies. Some clinical trials have shown that lipid-lowering therapies improve FMD, but still others report little to no effect [9,10]. Given these conflicting findings, a systematic review would be advantageous to gain a clearer understanding of the overall impact of lipid-lowering interventions on FMD. This meta-analysis was designed to evaluate the effectiveness of lipid-lowering drugs in improving FMD in patients at risk of cardiovascular disease. By pooling data from clinical studies, this analysis provides a more comprehensive assessment of the role of lipid-lowering therapies in enhancing endothelial function and potentially reducing cardiovascular risk. Additionally, we aimed to explore which class of lipid-lowering agents is more effective in protecting endothelial function.

## Methods

### Literature search strategy

This meta-analysis was conducted following the Cochrane Handbook guidelines to ensure methodological transparency and consistency. The study protocol was registered with PROSPERO (No: CRD42024597553), confirming that the research plan was established prior to the study. Furthermore, the reporting of results will strictly follow the Preferred Reporting Items for Systematic Reviews and Meta-Analyses checklist, attesting a comprehensive and transparent presentation of findings consistent with best practices.

In this meta-analysis, we performed an extensive literature search across major databases, including PubMed, Embase, and Web of Science, covering the period from 2011 to 2024. This search was specifically designed to identify RCTs evaluating the effects of lipid-lowering therapies on FMD. We combined keywords and Medical Subject Headings terms using Boolean operators, and the search was limited to publications in English. The terms used included “statins,” “PCSK9 inhibitors,” “ezetimibe,” and “FMD.” In addition, we manually searched for references in pertinent reviews to ensure a comprehensive dataset. The specific search terms are provided in the S1 File. The entire process of searching the literature, extracting data, and assessing quality was independently performed by two researchers working concurrently. In case of any discrepancies arising during data processing, we consulted a third researcher to achieve a consensus, thus ensuring that the methodology remains unbiased and transparent in accordance with the standards for systematic reviews.

### Inclusion and exclusion criteria

#### Inclusion criteria.

The criteria for inclusion of studies in this meta-analysis adhered to the PICOS framework.

1) P (Population): Human participants with no specific restrictions, including those at high risk for cardiovascular disease.
2) I (Intervention): On the basis of the control group, the patients were treated with lipid-lowering drugs.
3) C (Comparison): Conventional lipid-lowering therapy or no intervention was given.
4) O (Outcomes): At least one of the following outcomes: FMD, PWV, IMT, or LDL-C level.
5) S (Study Design): Parallel or crossover RCTs.

#### Exclusion criteria.

1) Animals were used as research objects;
2) Observational studies;
3) Articles with missing data or whose results were not available by October 1, 2024;
4) Literature type was review, commentary, meta-analysis, or case report;
5) The treatment cycle was < 4 weeks.

### Review of literature and data collection

Initially, two researchers meticulously reviewed and verified the literature based on the established inclusion and exclusion criteria. A final study was conducted to determine whether there were any objections. If there were any objections, a third researcher was invited to screen and discuss the retrieved literature to determine the final study. The data collected were as follows: 1) authors and year of publication; 2) sample size of the experimental and control groups; 3) follow-up time; 4) interventions; 5) outcome indicators; 6) drug type; 7) baseline diseases; and 8) outcomes: FMD, LDL-C, PWV, and IMT.

### Literature quality and risk of bias assessment

The quality of the included studies was assessed by two independent reviewers based on the criteria outlined in the Cochrane Handbook. The assessment focused on several critical aspects, and each criterion was categorized as “low, high,” or “unclear.”

### Data processing and analysis

To ensure consistency across studies and minimize discrepancies, we used the standardized mean difference (SMD) to evaluate the effect sizes. In crossover randomized controlled trials, data from the initial phase were extracted for evaluation. For studies with multiple measurement points, data collected at the completion of the trial were used in the final analysis.

Meta-analysis was performed using RevMan software version 5.4. Because all data were continuous variables, effect sizes were expressed as SMD with 95% CI. A P-value of <0.05 was deemed statistically prominent, with P < 0.01 indicating a higher level of significance. Statistical heterogeneity was assessed using P-values and I² statistics. If P < 0.10 and I² ≥ 50%, significant heterogeneity was indicated, which necessitated the application of a random effects model; otherwise, a fixed effects model was utilized. Subgroup analyses were performed according to the drug type, intervention duration, and disease type to identify potential sources of high heterogeneity. Publication bias was evaluated using funnel plots.

## Results

### Literature screening

**Fig 1:**
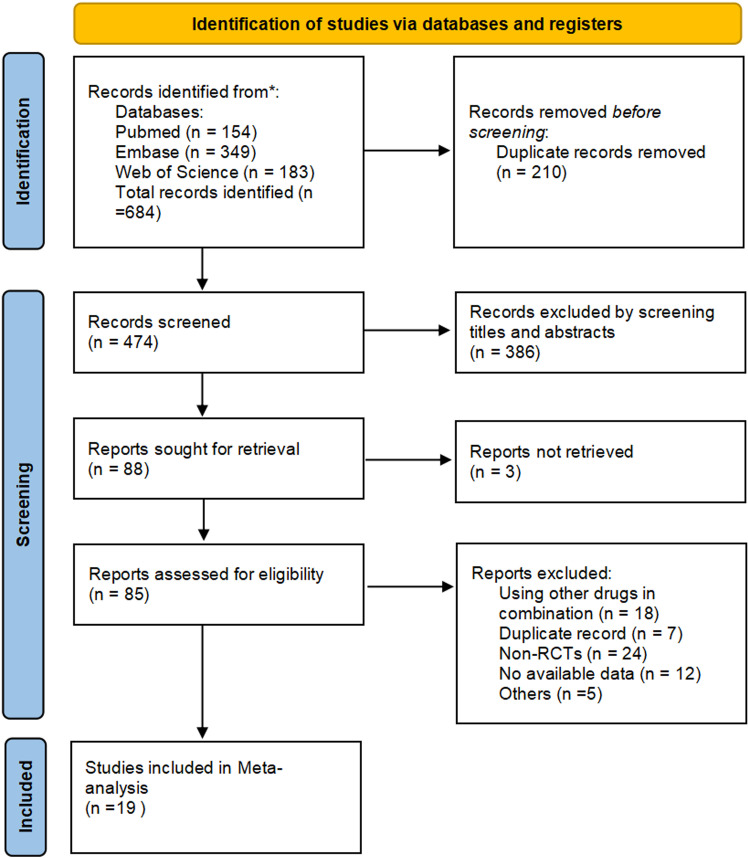
Study selection process for meta-analysis.

Our search yielded 684 articles, of which 210 duplicate articles were removed. After examining titles and abstracts, 386 articles were excluded. Additionally, three articles could not be retrieved. Further, 66 articles were eliminated for various reasons. All literature records are listed in S2 File. Ultimately, 19 articles were included in the meta-analysis (Fig 1).

### Elementary attributes and quality assessment

**Fig 2:**
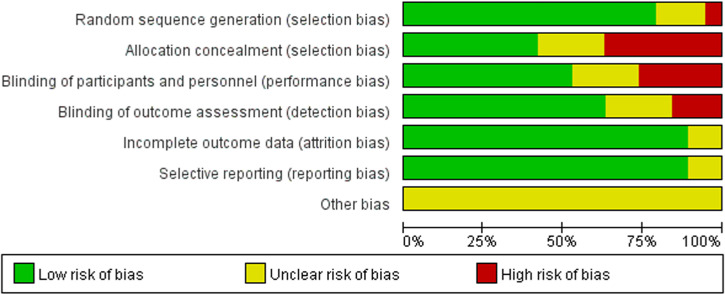
Visual summary of risk of bias.

**Fig 3:**
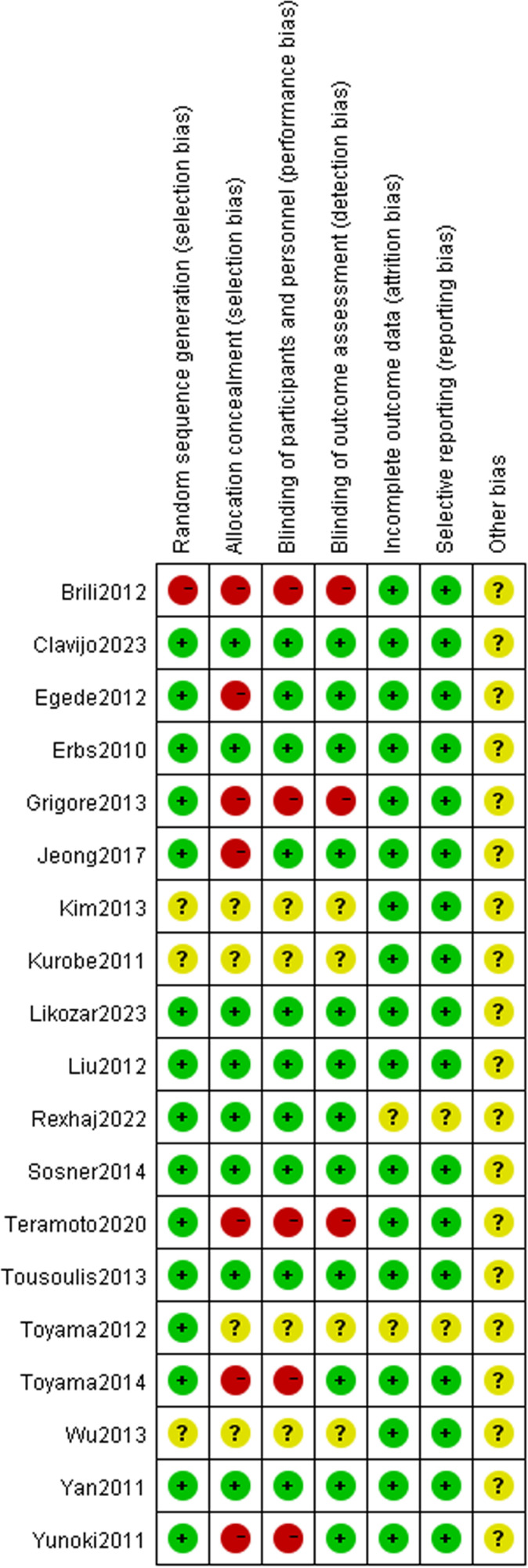
Risk of bias assessment chart.

All 19 included studies were RCTs [9,11–28], with a total of 1,004 participants. The average patient age was 28 and 67 years. Baseline diseases included hyperlipidemia, atherosclerotic cardiovascular disease (ASCVD), and heart failure (Table 1). The results of the quality assessment of the RCT are shown (Figs 2 and 3).

**Table 1:** Basic information about the included RCTs.

Author/Year	Participants		Disease	Group 1		Group 2		Treatment Duration	Outcome Measures
	Male (%)	Age (mean±SD)		Treatment	N	Treatment	N		
Sosner et al., 2014	54.55	56 ± 9/56 ± 9	Hyperlipidemia	Pravastatin 40 mg/d	11	Placebo	11	12 weeks	FMD, PWV, LDL-C, VO_2_ peak
Clavijo et al., 2023	NR	NR/NR	Peripheral Arterial Disease	Evolocumab 420 mg/2 weeks	35	Placebo	35	6 months	FMD, IMT
Toyama et al., 2012	NR	NR/NR	Coronary Heart Disease	Eicosapentaenoic Acid 1800 mg/d	29	Placebo	26	6 months	FMD
Teramoto et al., 2020	NR	58.5 ± 11.1/ 61.5 ± 7.9	Hyperlipidemia	Omega-3 4 g/d	15	Omega-3 2 g/d	15	8 weeks	FMD
Tousoulis et al., 2013	86	67 ± 12/ 67 ± 12	Heart Failure	Atorvastatin 40 mg/d	11	Atorvastatin 10 mg/d	11	4 weeks	FMD, LDL-C
Rexhaj et al., 2022	81.3	58.5 ± 9.7/58.5 ± 9.7	Coronary Heart Disease	Alirocumab 150 mg/d	68	Placebo	71	52 weeks	FMD, LDL-C
Egede et al., 2012	83.9	62.0 ± 9.9/ 60.0 ± 10.3	Coronary Heart Disease	Rosuvastatin 40 mg/d	43	Rosuvastatin 5 mg/d	44	12 months	FMD, LDL-C
Yan et al., 2011	40	57.56 ± 6.09/55.20 ± 8.35	Hyperlipidemia	Pitavastatin 2 mg/d	18	Pitavastatin 1 mg/d	18	8 weeks	FMD
Liu et al., 2012	62.5	58.2 ± 9.3/57.6 ± 9.5	Atherosclerosis	Rosuvastatin 40 mg/d	18	Rosuvastatin 20 mg/d	22	4 weeks	FMD, LDL-C
Grigore et al., 2013	100	52.4 ± 11.4/49.8 ± 8.6	Hyperlipidemia	Ezetimibe 10 mg/d	10	Placebo	10	6 weeks	FMD, LDL-C
Brili et al., 2012	50	31.4 ± 2.6/28.1 ± 2.0	Coronary Heart Disease	Atorvastatin 10 mg/d	17	Placebo	17	4 weeks	FMD
Kim et al., 2013	47.1	54.2 ± 12.5/52.6 ± 9.8	Coronary Heart Disease	Atorvastatin 40 mg/d	35	Atorvastatin 10 mg/d	35	6 months	FMD, IMT
Jeong et al., 2017	69.1	65.7 ± 8.97/62.4 ± 9.0	Diabetes	Pitavastatin 4 mg/d	35	Pitavastatin 1 mg/d	33	12 months	PWV
Wu et al., 2013	NR	NR/NR	Coronary Heart Disease	Simvastatin 20 mg/d	37	Placebo	18	8 weeks	FMD
Toyama et al., 2014	83.8	65.9 ± 8.2/68.7 ± 10.6	Hyperlipidemia	EPA 1800 mg/d	40	Placebo	40	3 months	FMD
Yunoki et al., 2011	85	38 ± 8/38 ± 8	Hyperlipidemia	Ezetimibe 10 mg/d	10	Placebo	10	4 weeks	FMD, LDL-C
Kurobe et al., 2011	82.5	66.2 ± 9.9/64.0 ± 14.1	Hyperlipidemia	Ezetimibe 10 mg/d	20	Placebo	20	3 months	FMD, PWV
Erbs et al., 2010	NR	NR/NR	Heart Failure	Rosuvastatin 40 mg/d	20	Placebo	20	12 weeks	VO_2_ peak, LDL-C
Likozar et al., 2023	NR	NR/NR	Coronary Heart Disease	Alirocumab 150 mg or Evolocumab 140 mg/2 weeks	38	Placebo	38	6 months	FMD, LDL-C

### Effect of lipid-lowering therapies on FMD

**Fig 4:**
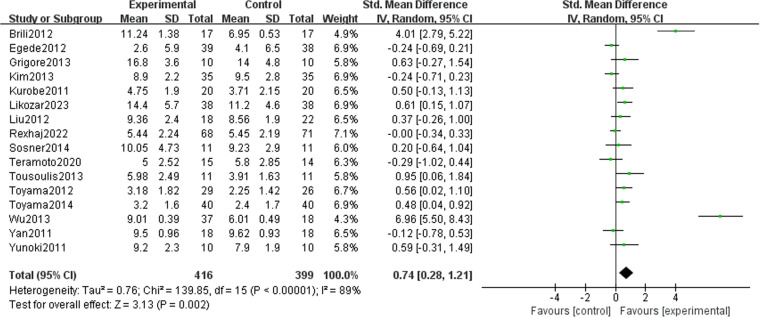
Meta-analysis of lipid-lowering therapies’ impact on FMD.

**Fig 5:**
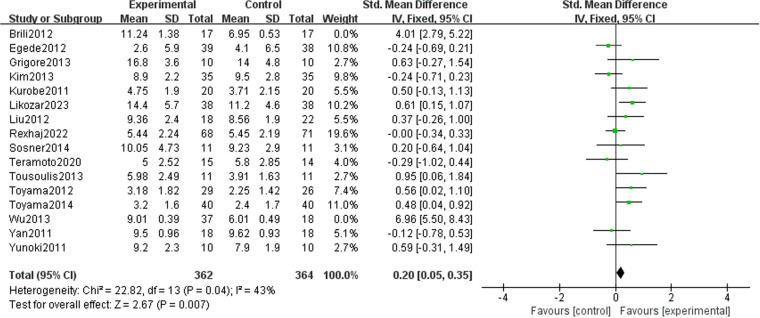
Meta-analysis of lipid-lowering therapies’ impact on FMD (post-exclusion).

**Fig 6:**
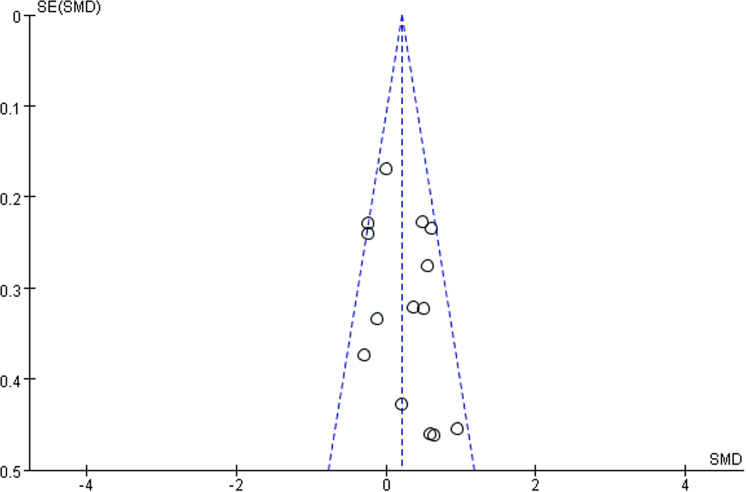
Funnel plot of FMD.

**Fig 7:**
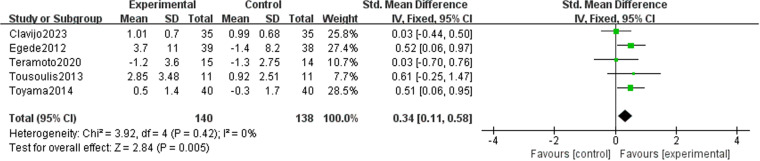
Meta-analysis of the effect of the mean change of lipid-lowering therapies on FMD: using literature reporting pre- and post-intervention differences.

We observed that lipid-lowering therapies were linked to a notable rise in FMD (0.74 [95% CI: 0.28, 1.21], P = 0.002, I^2 ^= 89%, 16 trials, 815 participants) (Fig 4). Owing to its considerable heterogeneity, a sensitivity analysis was performed with the exclusion of two studies, leading to a marked reduction in heterogeneity. The model was subsequently changed to a fixed-effect model, and the results remained statistically significant (0.20 [95% CI: 0.05, 0.35], P = 0.007, I^2^ = 43%, 14 trials, 726 participants) (Fig 5). A funnel plot after exclusion is shown in Fig 6. Some studies reported improvement in FMD by the difference between pre-and post-intervention; therefore, we conducted a meta-analysis of these studies, and the results also had statistical significance (0.34 [95% CI: 0.11, 0.58], P = 0.05, I^2 ^= 0%, 5 trials, 278 participants) (Fig 7).

### Effect of lipid-lowering therapies on LDL-C

**Fig 8:**
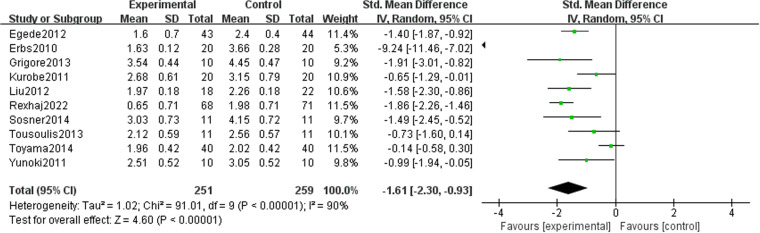
Meta-analysis of the effects of lipid-lowering treatments on LDL-C levels.

**Fig 9:**
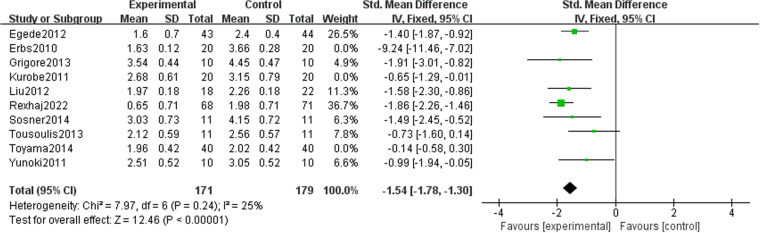
Meta-analysis of the effects of lipid-lowering treatments on LDL-C levels (post-exclusion).

**Fig 10:**
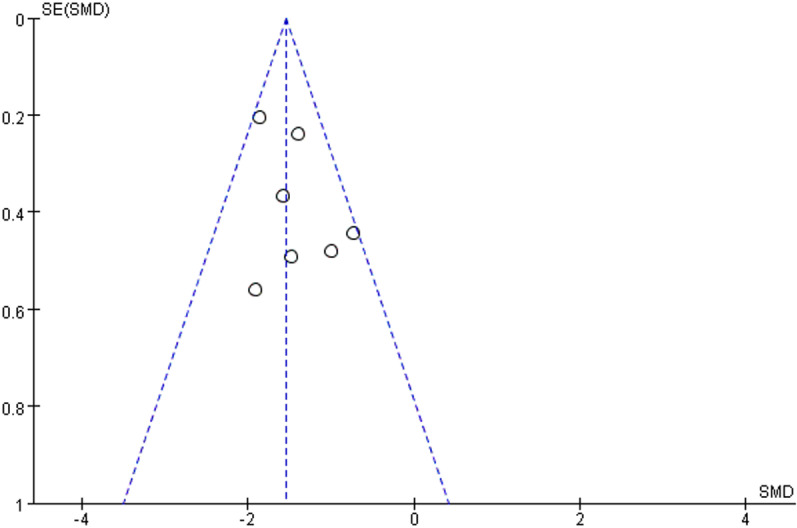
Funnel plot of LDL-C.

Our study revealed that LDL-C (−1.61 [95% CI: −2.30, −0.93], P < 0.00001, I^2 ^= 90%, 10 trials, 510 participants) was significantly reduced in the treatment group (Fig 8). Following the exclusion of three studies, the heterogeneity decreased, with I² = 25%, allowing for using a fixed-effects model in the analysis. The results indicated that the treatment group experienced superior outcomes compared to the control group (−1.54 [95% CI: −1.78, −1.30], P < 0.00001, I^2^ = 25%, seven trials, 350 participants), and the difference observed between the two groups was statistically significant (Fig 9). A funnel plot after exclusion is shown in Fig 10.

### Effect of lipid-lowering drugs on PWV

**Fig 11:**
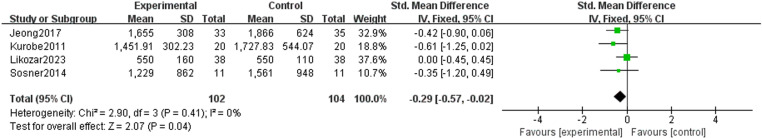
Meta-analysis of lipid-lowering therapies’ impact on PWV.

We observed a statistically significant reduction in PWV (−0.35 [95% CI: −0.57, −0.02], P = 0.04, I^2^ = 0.0%, four trials, 206 participants] with lipid-lowering therapy (Fig 11).

### Effect of lipid-lowering drugs on IMT

**Fig 12:**
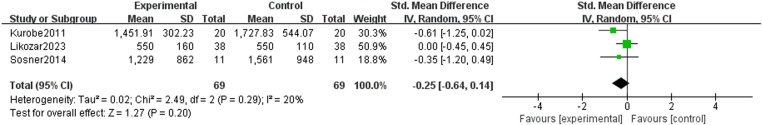
Meta-analysis of lipid-lowering therapies’ impact on IMT.

**Fig 13:**
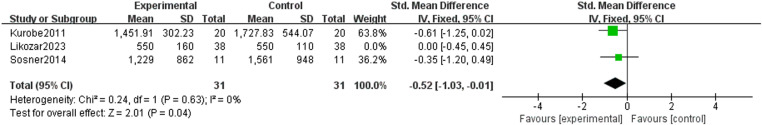
Meta-analysis of lipid-lowering therapies’ impact on IMT (post-exclusion).

After lipid-lowering drug intervention, IMT (−0.25 [95% CI: −0.64, 0.49], P = 0.2, I^2 ^= 20%, three trials, 138 participants) typically showed no significant change in the short term (Fig 12). When we excluded one study [28], there was a statistically significant difference in IMT (−0.35 [95% CI: −0.12, 0.49], P = 0.04, I^2^ = 0%, 2 trials, 62 participants) between the two groups, as shown in Fig 13.

### Effect of lipid-lowering therapies on VO_2_ peak

**Fig 14:**
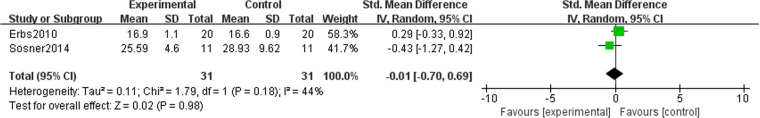
Meta-analysis of the effect of lipid-lowering therapies on VO_2_ peak.

There was no statistically significant reduction in PWV (−0.01 [95% CI: −0.70, 0.69], P = 0.98, I^2 ^= 44%, two trials, 62 participants) with lipid-lowering therapy (Fig 14).

### Subgroup analysis

**Fig 15:**
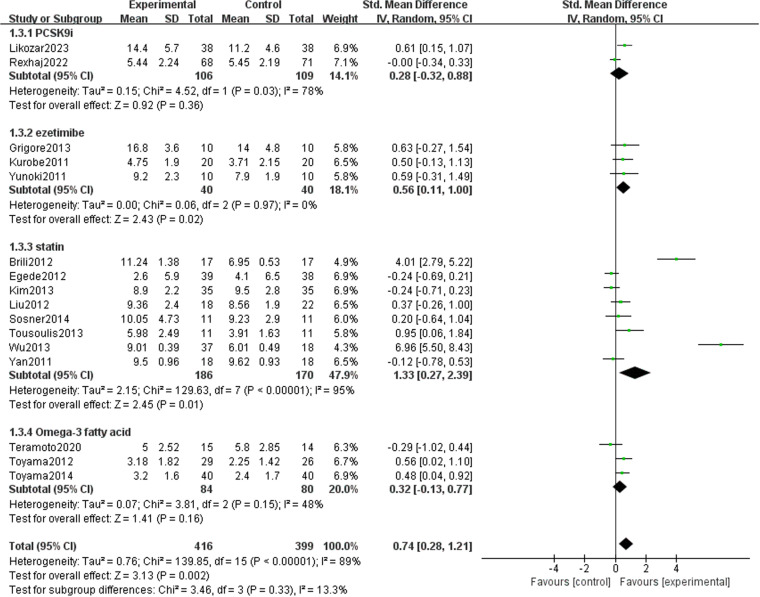
Subgroup analysis in drug types on FMD.

**Fig 16:**
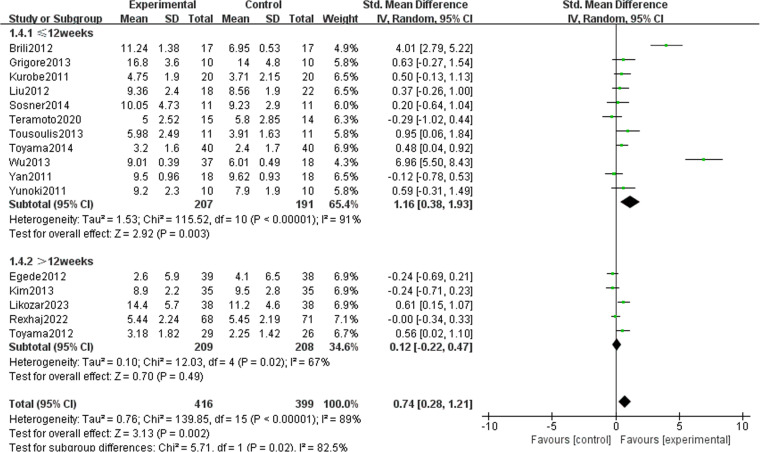
Subgroup analysis in intervention duration on FMD.

**Fig 17:**
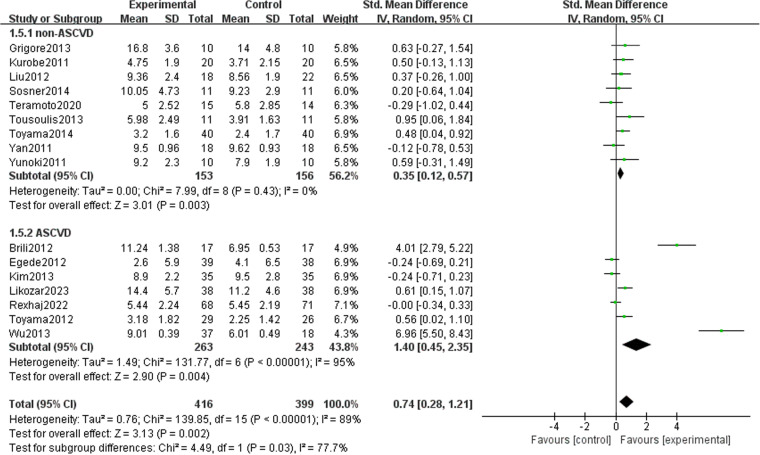
Subgroup analysis in underlying diseases on FMD.

To clarify the effect of various covariates on FMD, we conducted a subgroup analysis based on four key factors: drug type (Fig 15), intervention duration (Fig 16), and underlying diseases (Fig 17). A summary is table (Table 2). Among the different types of drugs, the intervention effects of statins (1.33 [95% CI: 0.27, 2.39], P = 0.01, I^2^ = 95%, eight trials, 356 participants) and ezetimibe (0.56 [95% CI: 0.11, 1.00], P = 0.02, I^2 ^= 0%, three trials, 80 participants) on FMD were statistically significant between the two groups. However, for PCSK9i and Omega-3 fatty acids, the results were not statistically significant, possibly because of the limited number of studies. In studies with no more than 12 weeks intervention duration, FMD (1.16 [95% CI: 0.38, 1.93], P = 0.003, I^2 ^= 93%, 11 trials, 398 participants) decreased significantly after drug intervention. Nevertheless, it might be that patients’ compliance in studies with longer intervention durations was lower; thus, there was no statistically significant difference between the two groups in studies with an intervention duration of >12 weeks. The improvement in FMD indicators by lipid-lowering drugs was obvious in both ASCVD (1.40 [95% CI: 0.45, 2.35], P = 0.004, I^2 ^= 95%, seven trials, 506 participants) and non-ASCVD cases (0.35 [95% CI: 0.12, 0.57], P = 0.003, I^2 ^= 0%, nine trials, 309 participants).

**Table 2:** Subgroup analysis of the included randomized controlled trials.

	Subgroups	Number	Combined Effect Value (95% CI) (%)	Heterogeneity I2 (%)	P
Overall		16	0.74 (0.28, 1.21)	89	0.002
Drug type	Statin	8	1.33 (−0.13, 0.77)	95	0.01
	PCSK9i	2	0.28 (−0.32, 0.88)	78	0.36
	Omega-3 fatty acid	3	0.32 (−0.13, 0.77)	48	0.16
	Ezetimibe	3	0.56 (0.11, 1.00)	0.0	0.02
Duration	≤12 weeks	11	1.16 (0.38, 1.93)	91	0.003
	>12 weeks	5	0.12 (−0.22, 0.47)	67	0.49
Underlying disease	Non-ASCVD	9	0.35 (0.12, 0.57)	0	0.003
	ASCVD	7	1.40 (0.45, 2.35)	95	0.004

ASCVD, atherosclerotic cardiovascular disease

### Publication bias

Publication bias was assessed using funnel plots. The funnel plots for FMD and LDL-C levels were not completely symmetrical, suggesting the possibility of publication bias.

## Discussion

In the present study, lipid-lowering agents were significantly more effective than the placebo in improving FMD. Because systematic reviews are based on small randomized clinical trials with substantial heterogeneity, evidence in this area is limited.

In analyzing the results from various studies, lipid-lowering medications exert a protective effect on endothelial function. This is particularly evident in high-risk patients such as those with ASCVD, where PCSK9 inhibitors, such as evolocumab and alirocumab, lead to significant improvements in endothelial function and reductions in plaque burden [29]. These drugs increase LDL availability and enhance LDL-C clearance, improving vascular health and FMD outcomes [30]. Longer intervention durations have also been shown to correlate with greater improvements in FMD, reflecting the cumulative benefits of sustained lipid-lowering. Studies such as the FOURIER trial [8] have highlighted that prolonged treatment with PCSK9 inhibitors alongside statins significantly reduces atherosclerotic progression, which may explain the enhanced FMD improvements observed over extended periods. Moreover, the vascular benefits of lipid-lowering therapies, including improvements in FMD, tend to be more pronounced compared to patients with isolated hyperlipidemia. This is likely owing to the more advanced vascular dysfunction in patients with coronary heart disease, which makes them more responsive to treatments that target both cholesterol levels and endothelial health [31]. Finally, as previous studies have shown, studies have suggested that higher doses [32,33] or combination therapies [34,35] (e.g., statins with PCSK9 inhibitors or ezetimibe) result in better FMD outcomes than standard-dose monotherapy. The combination of multiple lipid-lowering pathways, as observed in therapies combining statins with non-statin agents, has yielded synergistic effects [36–38], further improved vascular function and reducing atherosclerotic risk. These findings underscore the importance of both the type of lipid-lowering drug and treatment regimen in optimizing cardiovascular and endothelial health outcomes.

FMD, which is an indicator of vascular endothelial function, is an early marker of atherosclerosis. By measuring FMD, endothelial function damage can be detected early, which is helpful for early intervention and prevention of cardiovascular, cerebrovascular, and peripheral vascular diseases. Decreased FMD is closely associated with an increased risk of ASCVD. As endothelial dysfunction usually occurs in the early stages of these diseases, FMD can be used as an early indicator of atherosclerosis and cardiovascular events and can independently predict the risk of cardiovascular diseases. Similar to the present study, FMD has also been used to assess the effectiveness of cardiovascular disease interventions such as pharmacotherapy or lifestyle modification. It is strongly associated with systemic inflammation [39]. Chronic inflammation triggers or exacerbates endothelial dysfunction, and decreased FMD is usually accompanied by increased inflammatory markers. Therefore, FMD can indirectly reflect the inflammatory state of the body, especially in diseases such as atherosclerosis and metabolic syndrome, which often manifest as vascular failure to expand normally, enhanced inflammatory response, and increased thrombophilia [40].

FMD is closely associated with blood lipid levels. Excess LDL-C enters the vascular wall and is oxidized. Oxidized low-density lipoprotein (ox-LDL) has a direct damaging effect on endothelial cells, leading to increased inflammation, oxidative stress, and further damage to endothelial function. Therefore, reducing LDL-C levels helps reduce damage to the endothelium and improves vascular health. By lowering LDL-C levels, lipid deposition in the arterial wall, oxidative stress, and inflammatory damage to endothelial cells can be reduced, thereby promoting endothelial function recovery. Simultaneously, intensive lipid-lowering can also protect blood vessels through anti-inflammation, anti-oxidation, and nitric oxide production.

This systematic review and meta-analysis provides significant clinical utility by synthesizing data from 19 randomized controlled trials involving over 1000 patients, offering a comprehensive evaluation of the effects of various lipid-lowering therapies on endothelial function, as measured by FMD. The inclusion of diverse drug classes, including statins, PCSK9 inhibitors, ezetimibe, and omega-3 fatty acids, allows for a comparative assessment of their efficacy, which is particularly valuable for clinicians seeking to tailor treatment strategies based on patient-specific factors. Additionally, the use of FMD as a primary outcome provides a non-invasive, early marker of vascular health, enabling clinicians to identify and intervene in endothelial dysfunction before the onset of overt cardiovascular events. These findings reinforce the role of lipid-lowering therapies in improving vascular health and highlight their potential for early risk stratification and personalized treatment approaches.

## Conclusions

This study offers several notable clinical strengths that enhance its relevance and applicability in real-world practice. The rigorous methodology, including subgroup analyses based on drug type, intervention duration, and underlying diseases, provides nuanced insights into how different patient populations may respond to lipid-lowering therapies. This is especially important for high-risk groups, such as those with ASCVD, where endothelial dysfunction is a critical therapeutic target. By demonstrating the pleiotropic benefits of lipid-lowering agents beyond their cholesterol-lowering effects, this study contributes significantly to the growing body of evidence supporting their role in comprehensive cardiovascular care. These strengths underscore the importance of lipid-lowering therapies in improving vascular health and reducing cardiovascular risk.

The findings of the trials included in this study were heterogeneous, which is thought to be related to the selection of research subjects, sample sizes, and types and doses of drugs used in different studies. Therefore, a random-effects model was used to increase the reliability of the results. However, several limitations should be noted. First, small sample sizes in some subgroup analyses (e.g., PWV analysis with only 206 participants) may limit statistical power. Future studies should standardize designs and increase sample sizes to improve generalizability. Second, inconsistencies in drug types and dosages across studies may have contributed to outcome variability. Future trials should maintain consistency and explore dose-response relationships. Third, varying intervention durations and lack of long-term follow-up data prevent a comprehensive assessment of treatment effects. Future research should ensure consistent intervention periods and include long-term follow-up. Finally, reliance on FMD alone may not fully capture vascular health changes. Incorporating additional biomarkers (e.g., IMT, PWV) could provide a more comprehensive evaluation.

As a noninvasive method to assess arterial health, FMD has important clinical significance in early detection, risk prediction, efficacy evaluation of cardiovascular disease, and judgment of the effect of lifestyle interventions. Regular FMD monitoring can help identify early endothelial dysfunction and provide valuable evidence for personalized prevention and treatment. This will help reduce the incidence of cardiovascular disease and help patients better manage their existing cardiovascular risk factors.

In general, lipid-lowering therapy is not only the basic drug for the treatment of hyperlipidemia but also the cornerstone for the treatment of metabolic disorders such as diabetes and the prevention of adverse cardiovascular events. Their ability to modulate lipid profiles, protect endothelial function, and improve broader metabolic outcomes makes them an important component in the comprehensive care of patients with dyslipidemia and associated coexisting conditions.

## Supporting information

S1 File PRISMA checklist and search strategy. (DOCX)

S2 File Full list of screened records with inclusion/exclusion status. (XLSX)

## Abbreviations

FMD: flow-mediated dilation
RCT: randomized controlled trials
PWV: pulse wave velocity
LDL-C: low-density lipoprotein cholesterol
VO2 peak: peak O2 consumption
IMT: intimal media thickness
CIs: confidence intervals
NO: nitric oxide
SMD: standardized mean difference
ASCVD: atherosclerotic cardiovascular disease
ox-LDL: oxidized low-density lipoprotein
