# Supplementary material for: Effect of lipid-lowering therapies on flow-mediated dilation in patients: A systematic review and meta-analysis of clinical randomized controlled trials
Source: PLoS One. 2025 Jun 3;20(6):e0323210. doi: 10.1371/journal.pone.0323210 (PMC12132984; doi:10.1371/journal.pone.0323210)
Supplement: S1 File — (DOCX) [file pone.0323210.s001.docx]

| **Section and Topic** | **Item #** | **Checklist item** | **Location where item is reported** |
| --- | --- | --- | --- |
| **TITLE** | | |  |
| Title | 1 | The report is identified as a meta-analysis and systematic review. | Page 2 |
| **ABSTRACT** | | |  |
| Abstract | 2 | The abstract includes background, information sources, methods, total number of included studies and results for outcomes. | Page 2 |
| **INTRODUCTION** | | |  |
| Rationale | 3 | We described the rationale for the review in introduction. | Page 2 |
| Objectives | 4 | We provided an explicit statement of the objective in introduction. | Page 2 |
| **METHODS** | | |  |
| Eligibility criteria | 5 | We restricted the inclusion criteria based on the PICOS principle and described in Materials and Methods. | Page 4 |
| Information sources | 6 | We searched PubMed, Embase, and Web of Science, covering the period from 2011 to 2024.The search was specifically designed to identify randomized controlled trials (RCTs) that evaluated the effects of lipid-lowering therapies on FMD. | Page 4 |
| Search strategy | 7 | We provided search strategies in Supplementary data. | Supplementary data |
| Selection process | 8 | Two investigators independently reviewed each record and each report retrieved, and any disagreements were resolved through discussion. | Page 2 |
| Data collection process | 9 | Specify the methods used to collect data from reports, including how many reviewers collected data from each report, whether they worked independently, any processes for obtaining or confirming data from study investigators, and if applicable, details of automation tools used in the process. | Page 5 |
| Data items | 10a | List and define all outcomes for which data were sought. Specify whether all results that were compatible with each outcome domain in each study were sought (e.g. for all measures, time points, analyses), and if not, the methods used to decide which results to collect. | Page 5 |
|  | 10b | List and define all other variables for which data were sought (e.g. participant and intervention characteristics, funding sources). Describe any assumptions made about any missing or unclear information. | Page 5 |
| Study risk of bias assessment | 11 | Specify the methods used to assess risk of bias in the included studies, including details of the tool(s) used, how many reviewers assessed each study and whether they worked independently, and if applicable, details of automation tools used in the process. | Page 5 |
| Effect measures | 12 | Specify for each outcome the effect measure(s) (e.g. risk ratio, mean difference) used in the synthesis or presentation of results. | Page 5 |
| Synthesis methods | 13a | Describe the processes used to decide which studies were eligible for each synthesis (e.g. tabulating the study intervention characteristics and comparing against the planned groups for each synthesis (item #5)). | Table 1 |
|  | 13b | Describe any methods required to prepare the data for presentation or synthesis, such as handling of missing summary statistics, or data conversions. | Page 6 |
|  | 13c | Describe any methods used to tabulate or visually display results of individual studies and syntheses. | Page 6 |
|  | 13d | Describe any methods used to synthesize results and provide a rationale for the choice(s). If meta-analysis was performed, describe the model(s), method(s) to identify the presence and extent of statistical heterogeneity, and software package(s) used. | Page 6 |
|  | 13e | Describe any methods used to explore possible causes of heterogeneity among study results (e.g. subgroup analysis, meta-regression). | Page 7 |
|  | 13f | Describe any sensitivity analyses conducted to assess robustness of the synthesized results. | Page 6 |
| Reporting bias assessment | 14 | Describe any methods used to assess risk of bias due to missing results in a synthesis (arising from reporting biases). | Page 6 |
| Certainty assessment | 15 | Describe any methods used to assess certainty (or confidence) in the body of evidence for an outcome. | Page 6 |
| **RESULTS** | | |  |
| Study selection | 16a | Describe the results of the search and selection process, from the number of records identified in the search to the number of studies included in the review, ideally using a flow diagram. | Page 7 |
|  | 16b | Cite studies that might appear to meet the inclusion criteria, but which were excluded, and explain why they were excluded. | Page 7 |
| Study characteristics | 17 | The main characteristics of the included studies in the meta-analysis are described in Table 1. | Page 21 |
| Risk of bias in studies | 18 | Present assessments of risk of bias for each included study. | Page 23-24 |
| Results of individual studies | 19 | For all outcomes, present, for each study: (a) summary statistics for each group (where appropriate) and (b) an effect estimate and its precision (e.g. confidence/credible interval), ideally using structured tables or plots. | Page 24-28 |
| Results of syntheses | 20a | For each synthesis, briefly summarise the characteristics and risk of bias among contributing studies. | Page 8-9 |
|  | 20b | Present results of all statistical syntheses conducted. If meta-analysis was done, present for each the summary estimate and its precision (e.g. confidence/credible interval) and measures of statistical heterogeneity. If comparing groups, describe the direction of the effect. | Page 8-9 |
|  | 20c | Present results of all investigations of possible causes of heterogeneity among study results. | Page 8-9 |
|  | 20d | Present results of all sensitivity analyses conducted to assess the robustness of the synthesized results. | Page 8-9 |
| Reporting biases | 21 | Present assessments of risk of bias due to missing results (arising from reporting biases) for each synthesis assessed. | Page 23 |
| Certainty of evidence | 22 | Present assessments of confidence in the body of evidence for each outcome assessed. | Page 8-9 |
| **DISCUSSION** | | |  |
| Discussion | 23a | We provided a general interpretation of the results in the context of other evidence. | Page 10 |
|  | 23b | We discuss any limitations of the evidence included in the review. | Page 10 |
|  | 23c | We discuss any limitations of the review processes used in discussion part. | Page 10 |
|  | 23d | We discuss implications of the results for practice, policy, and future research in discussion part. | Page 10 |
| **OTHER INFORMATION** | | |  |
| Registration and protocol | 24a | The study protocol has been registered with PROSPERO (No: CRD42024597553) by Wang Xinyue. | Page 4 |
|  | 24b | We registered the trial with PROSPERO but did not write the protocol. | Page 4 |
|  | 24c | Our research is consistent with registration. | Page 4 |
| Support | 25 | We present detailed sources of Funding in the Funding section. | Page 13 |
| Competing interests | 26 | We declare that there are no conflicts of interest. | Page 13 |
| Availability of data, code and other materials | 27 | All data and images and tables not shown are presented in the supplementary materials. | supplementary materials |

*From:*  Page MJ, McKenzie JE, Bossuyt PM, Boutron I, Hoffmann TC, Mulrow CD, et al. The PRISMA 2020 statement: an updated guideline for reporting systematic reviews. BMJ 2021;372:n71. doi: 10.1136/bmj.n71

Search strategy in PubMed:

(("HMG-CoA Reductase Inhibitors"[MeSH Terms] OR ("Statin"[MeSH Terms] OR"statin*"[Title/Abstract]) OR ("Lovastatin"[MeSH Terms] OR "lovastatin*"[Title/Abstract]) OR ("Pravastatin"[MeSH Terms] OR "pravastatin*"[Title/Abstract]) OR ("Simvastatin"[MeSH Terms] OR "simvastatin*"[Title/Abstract]) OR ("Fluvastatin"[MeSH Terms] OR "fluvastatin*"[Title/Abstract]) OR ("Atorvastatin"[MeSH Terms] OR "atorvastatin*"[Title/Abstract]) OR ("Rosuvastatin"[MeSH Terms] OR "rosuvastatin*"[Title/Abstract]) OR ("Pitavastatin"[MeSH Terms] OR "pitavastatin*"[Title/Abstract]) OR ("Cholesterol Absorption Inhibitors"[MeSH Terms] OR "cholesterol absorption inhibitors"[Title/Abstract]) OR ("Ezetimibe"[MeSH Terms] OR "ezetimibe*"[Title/Abstract]) OR ("Proprotein Convertase Subtilisin/Kexin Type 9 Inhibitors"[MeSH terms] OR "PCSK9*"[Title/Abstract] OR("Evolocumab"[MeSH Terms] OR "evolocumab*"[Title/Abstract]) OR ("Alirocumab"[MeSH Terms] OR "alirocumab*"[Title/Abstract]) OR ("Inclisiran"[MeSH Terms] OR "inclisiran*"[Title/Abstract])) AND ("Flow-Mediated Dilation"[MeSH Terms] OR "flow-mediated dilation"[Title/Abstract] OR "FMD"[Title/Abstract])

Search strategy in Web of Science:

(HMG-CoA Reductase Inhibitors OR Statin OR Lovastatin OR Pravastatin OR Simvastatin OR Fluvastatin OR Atorvastatin OR Rosuvastatin OR Pitavastatin OR Cholesterol Absorption Inhibitors OR Ezetimibe OR Proprotein Convertase Subtilisin/Kexin Type 9 OR PCSK9 OR Evolocumab OR Alirocumab OR Inclisiran) and (Flow-Mediated Dilation OR FMD) and Article (Document Types) and English (Languages) Editions: WOS.SCI

Search strategy in EMBASE:

('hmg-coa reductase inhibitors':ab,ti OR 'statin':ab,ti OR 'lovastatin':ab,ti OR 'pravastatin':ab,ti OR 'simvastatin':ab,ti OR 'fluvastatin':ab,ti OR 'atorvastatin':ab,ti OR 'rosuvastatin':ab,ti OR 'pitavastatin':ab,ti OR 'cholesterol absorption inhibitors':ab,ti OR 'ezetimibe':ab,ti OR 'proprotein convertase subtilisin/kexin type 9':ab,ti OR 'pcsk9':ab,ti OR 'evolocumab':ab,ti OR 'alirocumab':ab,ti OR 'inclisiran'/exp OR 'inclisiran') AND ('flow-mediated dilation':ab,ti OR 'fmd'
